# Redefining uUTI therapy: Orlynvah as a promising oral alternative (sulopenem etzadroxil and probenecid)

**DOI:** 10.1097/MS9.0000000000003933

**Published:** 2025-09-23

**Authors:** Kamran Shahid, Syeda Rabiah Shahid, Maliha Khalid, Aminath Waafira

**Affiliations:** a Department of Medicine, Shaikh Khalifa Bin Zayed Al-Nahyan Medical and Dental College,Lahore, Pakistan; b Department of Medicine, Jinnah Sindh Medical University, Karachi, Pakistan; c Department of Medicine, The Maldives National University, Malé, Maldives

**Keywords:** antimicrobial resistance, FDA approval, uncomplicated urinary tract infections (uUTIs)

## Abstract

On December 24, the U.S. Food and Drug Administration approved Orlynvah (sulopenem etzadroxil, Iterum Therapeutics) as the first oral carbapenem for the treatment of uncomplicated urinary tract infections (uUTIs) in adult women. uUTIs, commonly caused by *Escherichia coli*, are limited to women without underlying comorbidities or anatomical abnormalities. Orlynvah, a prodrug hydrolyzed to sulopenem, exhibits broad-spectrum activity, including against multidrug-resistant and extended-spectrum β-lactamase-producing Enterobacteriaceae. The efficacy of Orlynvah was established through two large multicenter trials, which demonstrated superior outcomes compared to amoxicillin–clavulanate and improved efficacy over ciprofloxacin in cases involving resistant pathogens. The drug was generally well-tolerated, with few serious adverse events reported. Orlynvah offers a promising oral alternative in the era of rising antimicrobial resistance and represents a significant advancement in the management of uUTIs.

On December 24, The U.S. Food and Drug Administration (FDA) approved Orlynvah [sulopenem etzadroxil (SE), Iterum therapeutics] for the treatment of uncomplicated urinary tract infections (uUTI) attributed to certain bacteria, most notably *Escherichia coli*, in adult women, becoming the first oral carbapenem endorsed for the disease^[1]^.

uUTIs are infections afflicting the bladder or the lower urinary tract in women without any prior comorbidities or anatomical abnormalities, hence being inapplicable to UTIs in men, immunocompromised patients, or those with fever, obstruction, diabetes, or catheters^[1]^. It clinically presents as increased frequency and urgency of urination, suprapubic discomfort, and pain^[2]^.

An oral prodrug, hydrolyzed into sulopenem by the action of intestinal esterases, sulopenem etzadroxil, displays potent activity against a wide spectrum of pathogens including multidrug-resistant, extended-spectrum β-lactamase-producing enterobactericiae, a rising concern in the clinical management of UTIs ^[3,4]^. Sulopenem’s antibacterial effect, i.e., the inhibition bacterial cell wall formation is further augmented by the probenecid component of the drug which reduces its clearance, thereby increasing not only the efficacy but also the duration of its action^[5]^.

**HIGHLIGHTS**

- The FDA approved Orlynvah (sulopenem etzadroxil) as the first oral carbapenem for the treatment of uncomplicated urinary tract infections (uUTIs) in adult women.
- Clinical trials showed Orlynvah was more effective than amoxicillin–clavulanate and ciprofloxacin in patients with drug-resistant *Escherichia coli*.
- The drug was generally well-tolerated, with minimal serious adverse events, offering a promising and safe oral alternative for uUTI treatment.

This FDA approval follows positive outcomes seen in two multicenter clinical trials conducted across the U.S., Ukraine, and Russia, involving 3861 female patients^[1]^. The trial underscored marked superiority of Orlynvah to the comparator amoxicillin–clavulanate among patients with bacteria sensitive to the reference drug in their urine (61.7% for Orlynvah as compared to only 55.0 for the comparator)^[1]^. It displayed a slightly decreased potency relative to ciprofloxacin among patients with bacteria sensitive to the quinolone, but in individuals with resistant bacteria, Orlynvah once again proved more effective (48.1% compared to 32.9% for ciprofloxacin)^[1]^. The drug represents a substitute to the currently established first-line treatments of uUTIs, these being fosfomycin and nitrofurantoin^[6]^. Finding alternative treatments was necessary to avoid the long-standing problem of antibiotic resistance development, with bacteria like *E. coli* already demonstrating the ability to adapt to fosfomycin rapidly *in vitro*^[7]^. Meanwhile, an assessment of *in vitro* activity of sulopenem against *E. coli* demonstrated potent activity unhindered by resistance to trimethoprim–sulfamethoxazole and ciprofloxacin, even including the multidrug-resistant bacterial strains^[8]^. Nitrofurantoin being contraindicated during the last trimester of pregnancy, as well as causing jaundice and other hepatic problems, presented another motive for developing Orlynvah^[9]^.

However, the trials for Orlynvah did have a few limitations. First, only adult nonpregnant females were included, making the results not generalizable to broader populations^[10]^. Second, the success rates, while meeting the non-inferior criteria were still overall low, being 61.7% versus 55.0% for amoxicillin clavulante and 48.1% versus 32.9% for ciprofloxacin^[1]^. The primary end point, that is, both clinical resolution and microbiological eradication being used as the benchmark meant that patients in whom asymptomatic and thus clinically irrelevant bacteria persisted were counted as failures^[10]^. Both trials also only lasted for a total of 12 days each, which is hardly enough time to obtain an accurate appraisal of the drug’s risks of relapse or resistance emergence^[10,11]^. News about further trials demonstrating its safety in other populations, or its efficacy compared to nitrofurantoin or fosfomycin is yet to be announced.

Generally, the drug was well-tolerated, and serious adverse events were rare. The common side effects observed during the trials ranged from headache, nausea, vomiting, and diarrhea to vaginal yeast infections. Among particularly susceptible subjects, it may cause severe hypersensitivity reactions, thus the drug being strictly off-limits for any individuals with a known allergy to any of the constituents of the drug or any other β-lactams^[5]^. Although diarrhea is a common side-effect of the drug, concerns of developing a *Clostridium difficile* infection are present, with patients that develop severe watery or bloody stools being instructed to promptly contact their physician^[5]^. Furthermore, people with known hematological disorders, uric acid kidney stones, gout, or known allergies to any of the components of the drug – sulopenem etzadroxil and probenecid – should not take the medication^[5]^. It has also shown drug interactions with ketoprofen and ketorolac tromethamine, and so their concomitant use is prohibited^[5]^. Being a broad-spectrum drug and thus being at risk for development of resistance, it is recommended for treatment only when the vulnerable bacteria is either detected or strongly suspected to be the underlying cause of the infection^[12]^. Orlynvah is also reported to be substantially more expensive that the currently available therapies, leading to issues regarding affordability probably restricting its use to just cases of multidrug-resistant bacteria^[13]^.

Orlynvah has a unique mechanism of action and gives a promising alternative to existing treatments. It is quite effective and well-tolerated in clinical trials, with minimal side effects^[1,5]^. The emergence of a new antimicrobial resistance every other day, and the prevalence of uncomplicated UTIs, make Orlynvah a wonder drug. The approval of this drug represents a major advancement in urology, not only in the treatment of uncomplicated UTIs but also in addressing the issue of antibiotic resistance. Our work is in line with the TITAN Guidelines on the need for transparency in AI use in health care^[14]^.

## Data Availability

No data were generated for this manuscript.

## Research registration unique identifying number (UIN)

Name of the registry: N/A; Unique Identifying number or registration ID: N/A; Hyperlink to your specific registration (must be publicly accessible and will be checked): N/A.
